# Fully-automated system for dissolution rate of solid oral dosage forms according to the paddle method

**DOI:** 10.1155/S1463924693000239

**Published:** 1993

**Authors:** Erich Lamparter, Dieter Riedl

**Affiliations:** Department of Pharmaceutical Development Boehringer Ingelheim KG Ingelheim am Rhein D55216 Germany

## Abstract

This paper describes a fully-automated system (AUTO DISS^®^) for the determination of active ingredient release of solid oral dosage forms according to the paddle method of the US Pharmacopoeia (USP) and European Pharmacopoeia.

Twenty batches can be tested continuously, with the six individuals (tablets, capsules etc.) of one batch being examined synchronous. The components of the AUTO DISS^®^ system are presented and the operating steps of automatic filling with dissolution medium, dropping in of tablets, sampling and cleaning of vessels are described. Suitability for testing controlled-release drugs by means of automated buffer change from simulated gastric fluid to simulated intestinal fluid according to USP is also demonstrated. On-line determination of active ingredient concentration, as well as evaluation and documentation of measured values, is possible using an integrated automatic sampler in combination with various measuring instruments.

The AUTO DISS^®^ system is shown to be both rugged and accurate.


Journal of Automatic Chemistry, Vol. 15, No. 5 (September-October 1993), pp. 171-176

### Fully-automated system for dissolution rate of solid oral dosage forms according to the paddle method

**Erich Lamparter and Dieter Riedl**
*Department of Pharmaceutical Development, Boehringer Ingelheim KG, D55216 Ingelheim am Rhein, Germany*

*This paper describes a fully-automated system (AUTO DISS®) for the determination of active ingredient release of solid oral dosage forms according to the paddle method of the US Pharmacopoeia (USP) and European Pharmacopoeia.*

*Twenty batches can be tested continuously, with the six individuals (tablets, capsules etc.) of one batch being examined synchronously. The components of the AUTO DISS® system are presented and the operating steps of automatic filling with dissolution medium, dropping in of tablets, sampling and cleaning of vessels are described. Suitability for testing controlled-release drugs by means of automated buffer change from simulated gastric fluid to simulated intestinal fluid according to USP is also demonstrated. On-line determination of active ingredient concentration, as well as evaluation and documentation of measured values, is possible using an integrated automatic sampler in combination with various measuring instruments.*

*The AUTO DISS® system is shown to be both rugged and accurate.*

### Introduction

Dissolution testing of solid oral dosage forms plays an important part in:

(1) The development of new products, especially for optimizing the bioavailability of a drug substance.

(2) Stability testing, i.e. to detect changes in drug release as a result of different storage conditions.

(3) Quality control testing, i.e. to establish lot-to-lot equivalence of formulations.

The most widely used dissolution method is the paddle method, proposed by Levy and Hayes in 1960 [1] and modifed by Poole [2]. This test is an essential item in almost all pharmacopoeias. Data for registration at the CTA (Clinical Trial Application) and NDA (New Drug Application) stage must be included in the submitted documentation.

As the manual procedure of dissolution testing is expensive and time-consuming, researchers began proposing ways of automating the sampling and determination of the dissolved drug soon after the introduction of dissolution testing [3–9]. Sampling techniques are now highly advanced and complete installations are offered by vendors. These approaches to automating dissolution have reduced the time required for the test. The scope of automation, however, is restricted to testing a single batch. A fully automated system capable of analysing more than one batch must perform additional activities such as sampling, dropping of tablets into vessels, draining of dissolution medium, cleaning and drying of vessels mechanically.

Dissolution testing can be automated using laboratory robots [10–12]. These systems are now sophisticated enough to execute more complicated operational steps of the kind required in dissolution testing.

One serious disadvantage of robotic automation, however, is that all working steps have to be carried out sequentially, resulting in long processing times. Dissolution profiles with short sampling intervals are especially problematic. Moreover, because of their limited flexibility robotic systems are frequently ill-suited to processing smaller series of test samples involving a frequent change of method.

The present paper describes a more advanced system [13–15], it differs from laboratory robotic systems in that it can carry out all operating steps synchronously. The apparatus (AUTO DISS®, Manufacturer: Pharma Test, Apparatebau GmbH Siemens Strasse 5, D63512 Hainburg, Germany) is also suitable for fully automated testing of sustained-release dosage forms. This requires mechanically implemented change of buffer from simulated gastric fluid to simulated intestinal fluid according to USP.

### Concept

In drug release testing by the paddle method, the following basic operations can be combined into a single cycle:

(1) Filling of vessels with dissolution medium.

(2) Heating of medium to required temperature.

(3) Dropping of dosage forms into vessels.

(4) Adjustment of paddle speed.

(5) Sampling at predetermined intervals and transfer of solution to analytical instruments via autosampler.

(6) Change of dissolution medium from gastric fluid to intestinal fluid for sustained-release dosage forms by adding buffer solutions.

(7) Draining, rinsing and drying of dissolution vessels.

Upon completion of each separation step, the next cycle starts automatically in accordance with the preselected program.

Data collection and reduction, statistical calculation, report generation and presentation of data can be

0142-0453/93 $10.00 © 1993 Taylor & Francis Ltd.

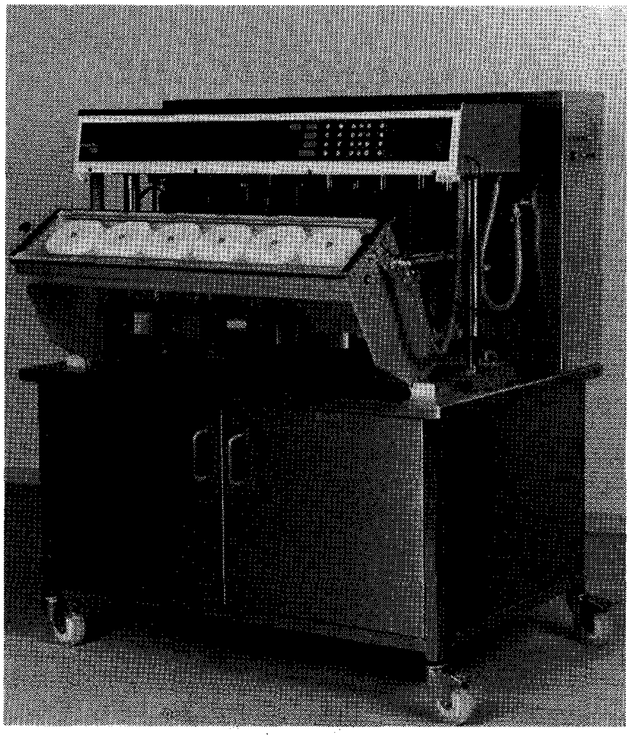

*Figure 1. The AUTO DISS system with dissolution tester, sample magazine, automatic filling system and draining system.*

implemented and a printout obtained via the PC-based controller after the analytical determination. Method and data management is performed on a data-base system.

### Components

The system comprises the following elements (see figure 1):

(1) Paddle apparatus with six vessels for tests and two vessels for blank and reference standard. The vessels and paddles are modified to allow draining of medium, rinsing and drying to be carried out automatically.

(2) Magazines for each vessel (under nitrogen) storing tablets or capsules for up to 20 batches.

(3) An automatic system equipped with syringes and valves for filling the vessels with dissolution medium, changing the pH of the medium (important for sustained-release dosage forms) and transferring the samples to an autosampler.

(4) A Gilson 232/401 (Gilson France, Villiers le Bel) autosampler for 360 samples, equipped with two Rheodyne valves for transferring test and standard solutions to HPLC, FIA, spectrophotometric or other instruments.

(5) Containers for storing dissolution medium and buffer solutions.

(6) A microprocessor (MS DOS, IBM compatible) to control the system and document the test parameters. Standard software, for entering and storing methods and data for evaluation and archiving. HPLC- and UV-software for transferring sample solutions from the sampler to the analytical instruments in on-line operation.

*Construction of vessel and paddle (figure 2)*

The dimensions of the vessel and paddle correspond precisely to those specified in the USP XXII.

A valve assembly consisting of the following parts is flange-mounted on the base of the dissolution vessel (1): a stainless steel screw thread (3) joined to the vessel and the screw-on valve assembly (4).

Permanently connected to part (4) is the guide bush (6) and (8) containing the freely movable Teflon plug (2) and steel spring (7). Also mounted on part (4) is the olive (5) as connector branch for a drain tube.

The stirrer consists of the paddle (11) which is welded to the metal tube (10). The paddle shaft (9) is connected to the channels (12) in the paddle.

Figure 2 also shows the upper end of the paddle shaft (9) and (10), which projects above the drive unit housing of the drug release apparatus (18). The metal housing (16) encases the paddle shaft and is fixed with the lock nut (17) and the paired seal rings (15), the upper shaft seal ring being connected to the screw fitting (14). The connecting branch (13) for water and compressed air supply is joined directly to the screw fitting (14).

*Sample magazine*

The sample magazine is installed above the front part of the drug release apparatus. This magazine consists of a stainless-steel plate with a 15 mm diameter hole for each dissolution vessel. The plate is provided with circular, rotatable Teflon disks with a diameter of 14 cm and a thickness of 16 mm.

Located around the periphery of each disk are 20 holes each with an internal diameter of 14 mm; these holes serve as containers for the dosage forms under test. With the aid of a motor, all six disks can be rotated synchronously so that the solid dosage form drops into the release medium when the hole in the disk is aligned directly above the hole in the plate. The sample magazine is protected against external influences by an acrylic glass hood. The magazine can also be aerated with air or nitrogen to prevent after-hardening of tablets or adhesion of soft gelatin capsules to the container wall due to steam from the heating bath during prolonged residence times.

*Dispenser station*

The dispenser station consists of eight syringe units which supply the sample vessels and the vessels for blank or reference standard solutions. The dilutors (50 ml volume), which are driven by the same motor, are used to fill the dissolution vessels with medium, withdraw samples and transfer the solutions to the automatic sampler, and to modify the pH of the dissolution medium when testing controlled release drugs. Each dilutor is connected to an eight-way valve. These valves allow media, such as buffer solutions or various release solutions, to be transferred to the vessels.

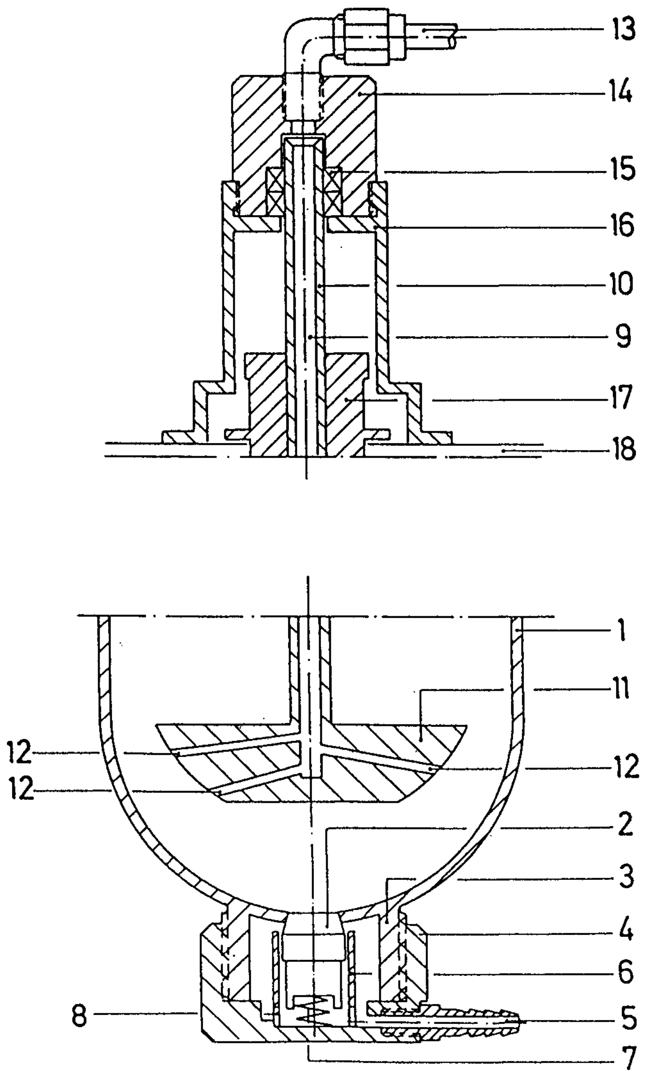

*Figure 2. Cross-section of glass vessel and paddle construction. (1) Dissolution vessel. (2) Plug. (3) Stainless steel screw thread. (4) Valve assembly. (5) Olive. (6) Guide bush. (7) Steel spring. (8) Guide bush. (9) Tube cavity. (10) Metal tube. (11) Paddle. (12) Channels. (13) Connecting branch for water and compressed air. (14) Screw fitting. (15) Seal rings. (16) Metal housing. (17) Lock nut. (18) Drive unit housing.*

### *Autosampler*

A Gilson 232/401 is preferred as autosampler, as it has a maximum capacity of 360 samples; it can also be used simultaneously as a sample injection system, as it has rheodyne valves. With the aid of the Gilson dilutor the collected test solutions can be transferred directly to the measuring instruments, such as spectrophotometer, high-performance liquid chromatograph or flow-injection system.

### *Controller*

The AUTO-DISS apparatus is controlled and monitored by a microprocessor (MS DOS, IBM compatible). Standard software is employed for entering and storing methods and data for evaluation and documentation. Software for on-line processing by means of Gilson 232/401

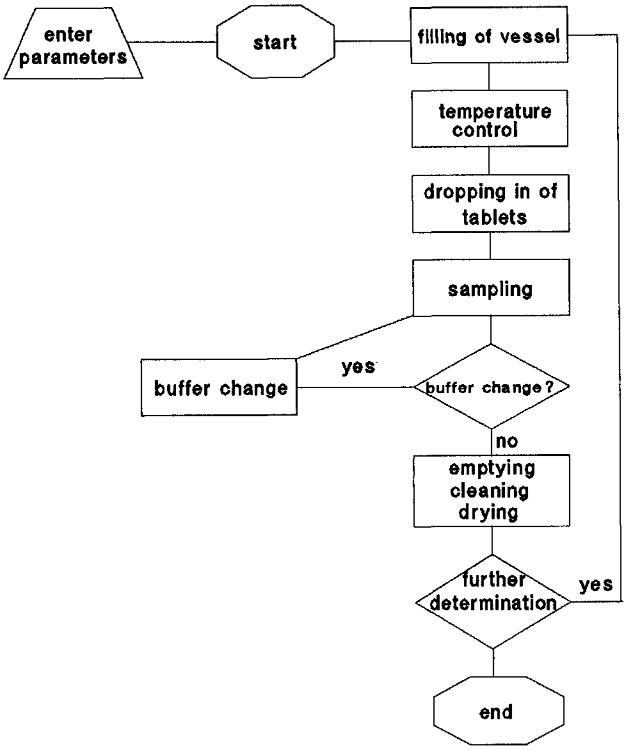

*Figure 3. Operating steps within a cycle.*

with spectrophotometric or liquid chromatographic evaluation is also available.

### Operating procedure

The operating procedure for one cycle, in which all the steps from filling the vessels with dissolution medium to cleaning and drying of the vessels are controlled automatically via a microcomputer, is shown in figure 3.

As many as 20 cycles can be processed automatically in succession, with up to six individual tablets being tested per cycle.

Before starting serial testing, parametrization is performed via the dialogue program. The following variables are established:

(1) Number of cycles (max $N = 20$, i.e. $20 \times 6$ tablets).

(2) Volume of dissolution medium (min 500 ml/max 1000 ml).

(3) Temperature of dissolution medium (min RT/max 45°C).

(4) Paddle speed (min 25 r.p.m./max 250 r.p.m.).

(5) Test solutions: volume (min 1 ml/max 50 ml), number and time of samplings.

(6) Rinsing times and rinsing volumes for sample tubes.

(7) Buffer solutions for pH change: volume dosing depending on type of buffer and required pH.

(8) Sample coding in autosampler system for identification for measurement and documentation of samples.

(9) Cleaning steps for vessels and paddles.

(10) Rinsing of syringe station and all tubes.

After establishing the parameters, the dissolution vessels are filled simultaneously with dissolution medium from a storage vessel by means of a syringe system. After a constant temperature has been reached, the microcomputer activates the drive motor for the sample magazine. When the motor has moved the sample magazine one step hole onward, it is switched off by the microcomputer. The samples to be tested fall simultaneously through specially positioned holes into the individual dissolution vessels. The paddles are then set in motion. The solutions to be measured are transferred according to a preselected program via the dispenser station directly to a Gilson 232 autosampler, or, optionally, to the multicell transport system of a spectrophotometer. For sustained-release preparations, the dispenser station also changes the pH buffer from simulated gastric fluid in all dissolution vessels simultaneously. In accordance with Method A of USP XXII, 250 ml quantities of buffer solution are pumped from storage containers through integrated valve circuits directly into the receiving vessels each containing 750 ml 0·1N hydrochloric acid. Automatic drainage of the dissolution medium, cleaning and drying of the vessels and paddles are described in detail in [13, 14]. After this cleaning step, the first cycle is completed and the dissolution vessels can be refilled with dissolution medium.

### Suitability and validation of the AUTO DISS system

To prove the suitability of the dissolution tester, a series of experiments was performed. In the following the results and experimental procedure according to USP XXII for dissolution testing of calibrator tablets are presented; buffer change experiments are then described which prove the suitability of the AUTO DISS system for testing the release of sustained-release dosage forms; and, finally, the robustness of the system is demonstrated by a 700 hour continuous test.

### *Dissolution of-prednisone and salicylic acid tablets*

*Prednisone tablets*

Test preparation: prednisone tablets 50 mg, lot J.

Test apparatus: AUTO DISS system, Pharma Test GmbH, Hainburg, Germany (apparatus II, Ph. Eur. V 5.4, apparatus I, USP XXII) with Gilson 232 autosampler system.

Test and measuring conditions: Dissolution medium: 900 ml water; paddle speed: 50 r.p.m. and 100 r.p.m., sampling interval: 30 min; sample volume: 5 ml; temperature: 37°C ± *0·5*°C; measurement method: flow injection analysis [14] followed by UV detection (242 nm).

Evaluation: The dissolved active ingredient concentrations were determined against prednisone USP XXII reference standard.

Specifications: 50 r.p.m.: 46–59%, 100 r.p.m.: 58–69%.

*Result*: prednisone in % (see table 1).

*Table 1. Results of dissolution of prednisone and salicylic acid.*

| | Prednisone (%) | | Salicylic acid (%) | |
|---|---|---|---|---|
| Tablet No. | 50 r.p.m. | 100 r.p.m. | 50 r.p.m. | 100 r.p.m. |
| 1 | 56·0 | 67·9 | 15·7 | 22·6 |
| 2 | 56·9 | 67·9 | 20·1 | 22·8 |
| 3 | 56·8 | 66·9 | 17·7 | 22·4 |
| 4 | 56·9 | 67·7 | 16·3 | 21·1 |
| 5 | 57·7 | 69·0 | 17·9 | 21·6 |
| 6 | 56·6 | 66·8 | 16·7 | 22·6 |
| Xm | 56·8 | 67·9 | 17·4 | 22·2 |
| s | 0·5 | 0·8 | 1·6 | 0·7 |
| RSD (%) | 1·0 | 1·5 | 9·0 | 3·0 |

*Salicylic acid tablets*

Test preparation: salicylic acid tablets 300 mg, lot H.

Test apparatus: AUTO DISS system, with Gilson 232 autosampler system.

Test and measuring conditions: Dissolution medium: 900 ml 0·05 mol/l phosphate buffer pH 7·4; paddle speed: 50 and 100 r.p.m., sampling interval: 30 min; sample volume: 5 ml; temperature: 37°C ± 0·5°C; measurement method: flow injection analysis followed by UV detection (296 nm) [14].

Evaluation: The dissolved active ingredient concentrations were determined against salicyclic acid USP XXII reference standard.

Specifications: 50 r.p.m.: 13–23%, 100 r.p.m.: 18–31%.

Result: salicyclic acid in % (see table 1).

*Results*: The active ingredient release specifications are complied with by the prednisone and the salicylic acid tablets, since all the individual values are within the specified tolerance limits. It is noticeable that the standard deviations within the test series show comparatively low values. The reproducibility of the tablet release profile is therefore good, and the dissolution vessels are very homogeneous in relation to each other.

### *pH change*

*In vitro* simulation of the conditions in the gastrointestinal tract, especially in testing of sustained-release or enteric dosage forms, requires a pH change from simulated gastric fluid to simulated intestinal fluid. These tests involving buffer change are very time-consuming and should therefore be automated. The AUTO DISS system was therefore designed to provide automated change of buffer, for which the following criterion must be met.

The US Pharmacopoeia stipulates a pH of 6·8 with a tolerance of ±0·05 pH units when changing the buffer from simulated gastric fluid (0·1 mol/l hydrochloric acid) to simulated intestinal fluid with the aid of phosphate buffer solutions. To ensure compliance with this narrow tolerance, the AUTO DISS system incorporates a highly precise dispenser station with additional switching valves for solvents for all dissolution vessels.

*Table 2. Reproducibility of buffer change from pH 1·0 to pH 6·8.*

| Vessel No. | pH 1st determination | 2nd determination | 3rd determination |
|---|---|---|---|
| 1 | 6·80 | 6·83 | 6·80 |
| 2 | 6·82 | 6·82 | 6·80 |
| 3 | 6·83 | 6·82 | 6·79 |
| 4 | 6·78 | 6·83 | 6·78 |
| 5 | 6·80 | 6·82 | 6·78 |
| 6 | 6·79 | 6·81 | 6·79 |
| Xm | 6·80 | 6·82 | 6·79 |
| s | 0·02 | 0·01 | 0·01 |
| RSD (%) | 0·3 | 0·1 | 0·1 |

*Experimental procedure and determination of precision*

A predetermined volume of 0·1 mol/l hydrochloric acid (750 ml) is transferred via the dispenser station to the vessels. To change the pH, the valve units are switched and 250 ml of 0·02 mol/l trisodium phosphate solution is drawn from a storage vessel and added to all the dissolution vessels simultaneously via the piston syringes. A rinsing cycle is then carried out to remove the excess buffer solution from the system.

Table 2 demonstrates the good reproducibility of the buffer change from simulated gastric fluid (pH 1) to simulated intestinal fluid (pH 6·8). A tolerance of ±0·05 pH units is maintained in all tests.

*Recording of a dissolution profile—theophylline sustained-release capsules*

Test preparation: Theophylline sustained-release capsules 300 mg, lot 801102. Manufacturer: Boehringer Ingelheim KG.

Test apparatus: AUTO DISS system with Gilson 232 autosampler system.

Test and measuring conditions: Dissolution medium: 900 ml 0·1 mol/l hydrochloric acid/phosphate buffer 6·8; paddle speed: 150 r.p.m., sampling intervals $t = 1, 2, 4, 6, 8, 10$ h; sample volume: 5 ml.

Buffer change from pH 1 to pH 6·8 is performed after 1 h.

Temperature: 37 ± *0·5*°C; measurement method UV detection (270 nm).

Evaluation: Determination of the dissolved active ingredient concentrations is performed against theophylline reference substance. The concentrations of theophylline calculated in the dissolution test are stated in % in table 3.

*Robustness of the AUTO DISS® system*

The AUTO DISS apparatus was subjected to a stress test in which the system was kept in continuous operation for about 700 hours. Analyses were performed both on development products with a low active ingredient content (pramipexole tablets 0·1 mg) and on licensed products of the Lonarid series (tablets with 500 mg paracetamol, 0·5 mg dihydroergotamine and 10 mg codeine phosphate). Altogether 526 batches and/or stability samples were tested during the investigational period ($N = 6$; 3156 samples).

As well as checking the reproducibility of the products (of known content uniformity), particular attention was paid to assessing the mechanical robustness of the AUTO DISS apparatus.

*Comment*

During the 700 hour long-term test, no mechanical deficiencies were observed, except for a rapidly repaired leak in one dilutor. All other mechanical moving parts, such as media drain valves, eight-way valves, dispenser unit and the Gilson 232/401 autosampler, worked perfectly. Neither were there problems with the sintered glass filters for the samples, problems of entrainment of active ingredients in the cleaning operations or problems of adsorption on tubing.

All the individual data recorded were within the specified requirements. The reproducibility of the groups of investigated products exhibited similar coefficients of variation as the content uniformity of the corresponding products. It can therefore be concluded that the entire system operates reproducibly.

The positive general impression obtained for the AUTO DISS apparatus allows the conclusion that the system is extremely reliable and therefore suitable for use in the development and quality-control areas.

### Discussion

Manual testing of active ingredient release from oral dosage forms using the paddle method is the most time-

*Table 3. Results of dissolution of theophylline from sustained-release capsules in % after 1, 2, 4, 6, 8, and 10 h.*

| Sampling intervals (h) | Active ingredient concentration (%) 1 | 2 | 3 | 4 | 5 | 6 | Xm | s | RSD (%) |
|---|---|---|---|---|---|---|---|---|---|
| 1 | 23·0 | 20·0 | 16·5 | 23·5 | 18·0 | 21·5 | 20·4 | 2·8 | 13·6 |
| 2 | 37·5 | 32·8 | 29·4 | 36·8 | 30·8 | 34·8 | 33·7 | 3·3 | 9·7 |
| 4 | 60·3 | 53·6 | 48·9 | 59·0 | 52·9 | 56·3 | 55·2 | 4·2 | 7·7 |
| 6 | 79·3 | 72·6 | 71·8 | 79·3 | 73·9 | 75·3 | 75·4 | 3·3 | 4·3 |
| 8 | 93·0 | 85·6 | 87·5 | 92·4 | 88·2 | 90·3 | 89·5 | 2·9 | 3·2 |
| 10 | 100·8 | 93·3 | 97·3 | 100·8 | 98·7 | 99·4 | 98·4 | 2·8 | 2·9 |

consuming operation in the analytical characterization of drugs. Filling, buffer change (for controlled-release drugs), emptying and cleaning operations in particular are mindless activities for laboratory staff. Automating these operations results not only in more streamlined performance of dissolution testing but also in standardization of techniques.

With the AUTO DISS system, as many as 20 determinations can be carried out in a 24-hour rhythm. This represents a considerable increase in capacity.

Combining the various analytical methods, for example spectrophotometry (including diode array spectroscopy), liquid chromatography and flow injection analysis with the AUTO DISS system increases the flexibility of the system. The requirement for laboratory staff is thereby reduced and technicians are relieved of time-consuming routine work.

### References

1. Levy, G. and Hayes, B. A., *New England Journal of Medicine*, **262** (1960), 1053.
2. Poole, J. W., *Drug Information Bulletin*, **3** (1969), 8.
3. Cioffi, F. J., Abdou, H. M. and Warren, A. T., *Journal of Pharmaceutical Science*, **65** (1976), 1234.
4. Wahlich, J. C. *Pharmaceutical Technology*, **3**, 34 (1980), 34.
5. Wahlich, J. C., *International Journal of Pharmacology*, **36** (1987), 175.
6. Dong, M. W. and Hockman, D. C., *Pharmaceutical Technology*, **11** (1987), 70.
7. Fahr, F., Kala, H., Wenzel, U., Welk, G. and Zessin, G., *Pharmazie*, **39** (1984), 49.
8. George, R. C., Cornelius, K. E. and Contrario, J. J., *American Laboratory* (Fairfield, Conn.), **20** (1988), 106.
9. Soltero, R., Robinson, J. and Adair, D., *Journal of Pharmaceutical Science*, **73** (1984), 799.
10. Papas, N. A., Alpert, M. Y., Marchese, S. M. and Fitzgerald, J. W., *Analytical Chemistry*, **57** (1985), 1408.
11. Gänshirt, H., Tessun, G. and Wolfshütz, R., *Pharmazeutische Industrie*, **47** (1985), 1063.
12. Kostek, L. J., Brown, B. A., Erhart, L. G. and Culey, J. E., in *Advances in Laboratory Automation: Robotics* (Zymark Corporation), 311.
13. Grady, L. T., *Pharmazeutische Industrie*, **45** (1983), 640.
14. Lamparter, E., Diederich, H. J., Eppelmann H., Linn, H. O. and Peil, H., *Pharmazeutische Industrie*, **49** (1987), 621–626.
15. Lamparter, E., Lunkenheimer, Ch., Seibel, U. and Voss, H., *Pharmazeutische Industrie*, **53** (1991), 277.
16. Lamparter, E. and Lunkenheimer, Ch., *Journal of Pharmaceutical and Biomedical Analysis*, **10** (1992), 727.

